# Rehabilitative game-based exercise integrated into conservative treatment for adolescent idiopathic scoliosis: a pilot randomized controlled trial

**DOI:** 10.1186/s13102-026-01981-6

**Published:** 2026-08-18

**Authors:** Hande Tunc, Devrim Tarakcı, Yasemin Koc, Eylul Pınar Kısa, Deniz Oke

**Affiliations:** 1 Graduate School of Health Sciences, Department of Physical Therapy and Rehabilitation, Istanbul Medipol University, Cibali Mah., Atatürk Bulvarı, No:27, 34083 Istanbul, Fatih Turkey; 2 Department of Biostatistics, Institute of Health Sciences, Ankara University, Ankara, Turkey; 3 Faculty of Health Sciences, Department of Occupational Therapy, Istanbul Medipol University, Istanbul, Turkey; 4 Department of Physical Medicine and Rehabilitation, University of Health Sciences Gaziosmanpasa Training and Research Hospital, Istanbul, Turkey

**Keywords:** Scoliosis, Exercise therapy, Gamification, Postural balance, Motivation

## Abstract

**Background:**

The incorporation of game-based exercise into conservative treatment for adolescent idiopathic scoliosis (AIS) has the potential to increase patient engagement, yet the evidence supporting its efficacy remains sparse. This study aimed to evaluate the effects and usability of game-based rehabilitative exercises in conservative treatment for AIS.

**Methods:**

This assessor- and statistician-blinded pilot randomized controlled trial involved female participants aged 10–14 years with AIS, Cobb angles of 25°–40°, and full-time Chêneau-type brace use. Twenty-five participants were randomly assigned to the control or rehabilitative game-based exercise group. Both groups received scoliosis-specific physiotherapy exercises, core stabilization, balance training, and full time bracing for 24 weeks. Starting from week 5, the game-based group participated in game-based exercises targeting core stabilization and balance, conducted both in clinic and at home. The primary outcomes were Cobb angle, posterior trunk symmetry, and perceived trunk deformity. Secondary outcomes included trunk rotation, motivation to participate in physical activity, balance, and system usability. A per-protocol analysis was conducted on 20 participants.

**Results:**

Both groups experienced a significant reduction in the thoracic Cobb angle, but a significant decrease in the lumbar Cobb angle was observed only in the game-based group. There were no significant differences between the groups in terms of changes in Cobb angle, trunk rotation, posterior trunk symmetry, perceived deformity, or balance. Motivation levels significantly declined in the control group but remained stable in the game-based group. After adjustment for baseline motivation, no significant between-group difference was observed at T1 (*p* = 0.207), whereas a significant difference favoring the game-based group was observed at T2 (*p* = 0.015, partial η^2^ₚ = 0.303). The median system usability score was 76.50, surpassing the threshold for acceptability, and no adverse events related to the intervention were reported.

**Conclusions:**

In this pilot sample, no statistically significant between-group differences were detected in radiological, trunk symmetry, perceived deformity, or balance outcomes. Motivation declined in the control group but was maintained in the game-based group, and the system demonstrated satisfactory usability. Larger, adequately powered trials are required to confirm these preliminary findings.

**Trial registration:**

ClinicalTrials.gov identifier NCT06469021 (Record date: 16/06/2024).

**Supplementary Information:**

The online version contains supplementary material available at https://doi.org/10.1186/s13102-026-01981-6.

## Introduction

Adolescent idiopathic scoliosis (AIS) is a three-dimensional spinal deformity that develops between 10 and 18 years of age without an identifiable neuromuscular, congenital, or syndromic cause. It is characterized by coronal curvature, vertebral rotation, and sagittal imbalance [1]. In AIS, deformity, postural asymmetry, balance problems impose considerable physical and psychosocial burdens on patients, partly through their adverse effects on cosmetic appearance [2, 3].

International guidelines for managing AIS recommend bracing and exercise approaches for mild-to-moderate curves. However, these approaches may be challenging for patients over the long term, and adherence to home exercise programs has been reported to decline over time [4]. Moreover, the inherent complexity of adolescence may exacerbate adherence problems, while the perception of conventional repetitive exercise methods as monotonous may increase the risk of exercise discontinuation [5].

Technology-assisted and gamified exercise applications are increasingly used to complement conventional exercise programs by providing real-time feedback, progressive tasks, and performance tracking [6, 7]. These features may be particularly relevant in AIS, where prolonged and repetitive home exercise can challenge motivation and sustained participation. Technology-assisted exercise may therefore be integrated as a complementary component of clinically prescribed therapeutic programs rather than used as a replacement.

Although existing literature provides promising findings regarding technology-assisted approaches in AIS rehabilitation, randomized controlled trials integrating rehabilitative game-based exercise into standard conservative treatment remain limited [8]. This pilot randomized controlled trial aimed to evaluate the preliminary effects of rehabilitative game-based exercise integrated into conservative AIS treatment on Cobb angle, trunk symmetry, body appearance, balance, and motivation to participate in physical activity, as well as the usability of the system employed.

## Methods

### Study design

**Fig. 1:**
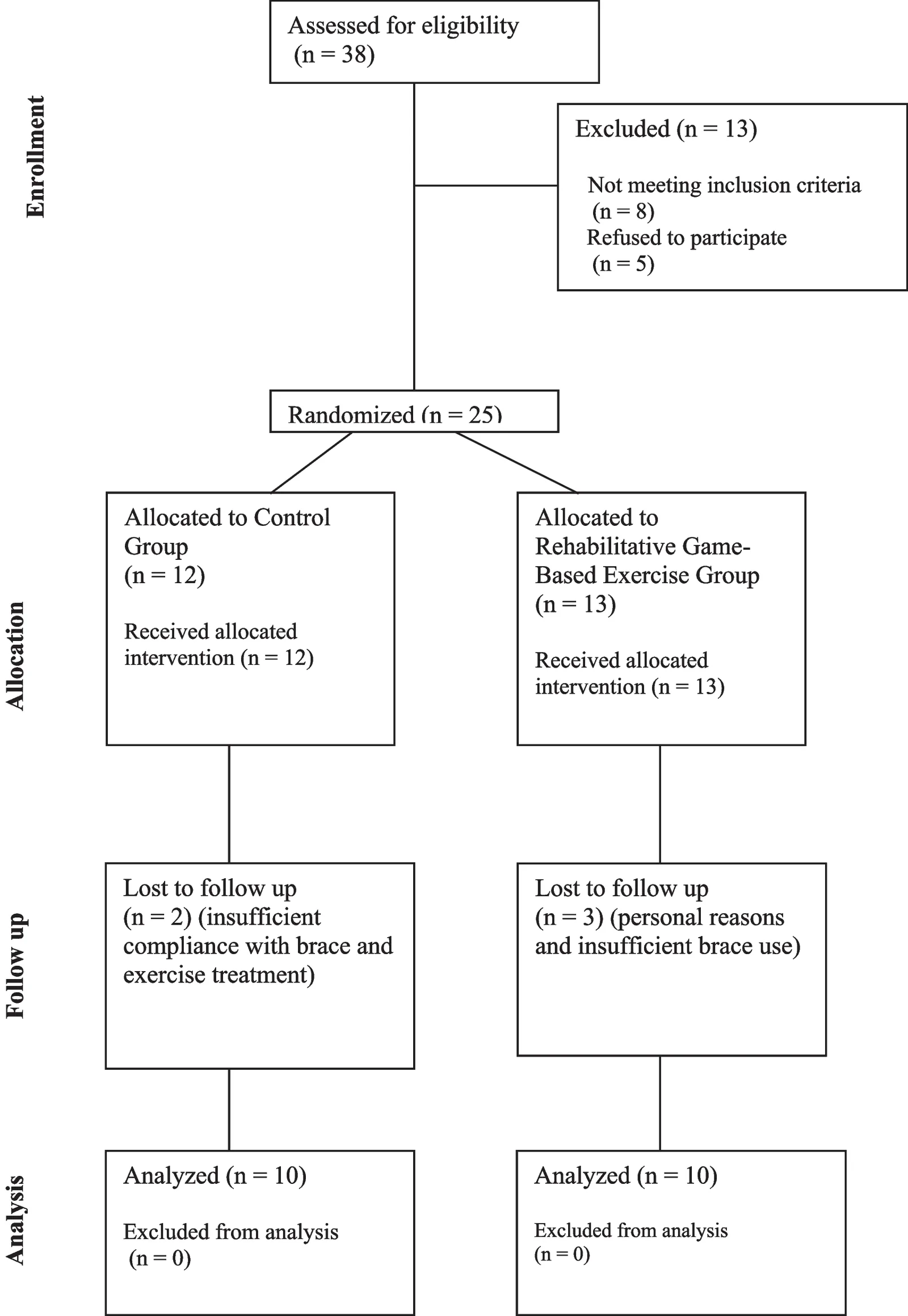
Consort flow diagram

This prospective, randomized controlled, assessor- and statistician-blinded pilot trial was designed to evaluate the feasibility and preliminary effects of a rehabilitative game-based exercise approach in female participants with AIS. Participants were randomly allocated to the Control Group (CG) and the Rehabilitative Game-Based Exercise Group (RGBEG) in a 1:1 ratio. Assessments were performed at baseline and at weeks 12 and 24. This pilot randomized controlled trial was conducted and reported in accordance with the CONSORT 2010 guidelines. The completed CONSORT checklist is provided as an additional file, and the participant flow diagram is presented in Fig. 1.

### Study setting

The study was conducted at the Aktif R&D Academy Consulting Center. Patients diagnosed with AIS who met the eligibility criteria were referred to the research team by a specialist physician working in the field of scoliosis. The study protocol was reviewed and approved by the Istanbul Medipol University Non-Interventional Clinical Research Ethics Committee (reference no. E-10840098–202.3.02–3224; approval no. 529). Participant recruitment began on 14 October 2024 and was completed on 9 September 2025. The final participant completed the 24-week follow-up assessment on 1 March 2026.

### Ethics and consent to participate

The study was conducted in accordance with the ethical principles of the Declaration of Helsinki. Before participation, all participants and their parents were provided with detailed information regarding the purpose, procedures, duration, potential risks, and voluntary nature of the study. As all participants were younger than 18 years, written informed consent to participate was obtained from the parent or legal guardian of each participant prior to enrolment.

### Participants

Skeletally immature female patients diagnosed with AIS by a physician and prescribed brace treatment were included. To minimize potential sex-related differences, the study was restricted to female participants. All participants used a three-dimensional rigid Chêneau-type brace designed according to their curve pattern on a full-time basis. In accordance with international guidelines, brace wear was prescribed for at least 20 h per day [1]. Brace adherence and biomechanical fit were evaluated during routine physician follow-up visits, and modifications were made when necessary to maintain optimal corrective effectiveness.

The inclusion criteria were a physician-confirmed diagnosis of AIS, female sex, age 10–14 years, a Cobb angle of 25°–40°, skeletal immaturity (Risser grade 0–2), and full-time use of a three-dimensional rigid Chêneau-type brace (≥ 20 h/day).

The exclusion criteria were previous spinal surgery, development of a surgical indication during the study, participation in any other structured exercise program, participation in competitive sports, cardiac or respiratory dysfunction, or any health condition that precluded participation in exercise.

Participants who withdrew, were lost to follow-up, did not comply with full-time brace wear, and/or attended fewer than 80% of the prescribed exercise sessions were not included in the per-protocol analysis. In both groups, brace and exercise adherence was monitored using a Brace and Exercise Diary developed by the researcher and verified by the families. In the RGBEG, completion of game-based home exercises was additionally verified using digital usage records obtained from the BeCure Virtual Clinic System.

### Sample size, randomization, and blinding

The sample size estimate was calculated using G*Power version 3.1.9.7 based on the study by Gür et al. [9]. A between-group difference of 10° in thoracic Cobb angle was considered clinically meaningful. With an alpha level of 0.05 and 80% power, the minimum number of participants required to complete the study was calculated to be 10 per group. To account for an anticipated 20% loss to follow-up during the 24-week intervention, the target sample size was set at 25 participants. Given the pilot nature of the study, this sample size was considered adequate for evaluating feasibility and preliminary clinical effects rather than providing definitive evidence of efficacy.

Before recruitment began, the random allocation sequence was created using www.randomizer.com by an independent staff member who was not involved in participant enrolment, assessment, intervention delivery, or data analysis. Eligibility screening and participant enrolment were performed by a physician experienced in scoliosis care. After completion of the baseline assessment, the principal investigator opened the sequentially numbered, opaque, sealed envelopes and assigned participants to either the CG or the RGBEG according to the pre-generated randomization sequence.

Because of the nature of the study, blinding of the participants and the physiotherapist delivering the intervention to group allocation was not possible. However, the physician who performed the radiological assessments and the statistician who conducted the statistical analyses were blinded to group allocation.

### Interventions

The intervention period was 24 weeks in both groups. All participants received full-time treatment with a three-dimensional rigid Chêneau-type brace for at least 20 h per day, PSSE based on the Barcelona Scoliosis Physical Therapy School (BSPTS) approach, core stabilization exercises, and balance training. The PSSE program followed the same structure and progression in both groups but was individualized according to each participant’s curve type. The only difference between the groups was the delivery mode of the core stabilization and balance components from week 5 onward: the RGBEG performed these components through game-based digital systems, whereas the CG continued with conventional exercises. It comprised three progressive levels—initial, intermediate, and advanced—each lasting 8 weeks. Within each level, participants performed 3 sets of 6 repetitions during the first 4 weeks and 4 sets of 8 repetitions during the subsequent 4 weeks. During the first 4 weeks of the intervention, core stabilization and balance exercises were delivered conventionally in both groups to facilitate correct transversus abdominis activation and neutral positioning of the spine and pelvis. The planned exercise frequency and session duration were matched between groups.

### Control group

Participants in the CG performed conventional core stabilization and balance exercises throughout the 24-week intervention period. The exercises were progressively advanced according to the predefined treatment protocol and each participant’s performance. Detailed information regarding the exercise content and progression is provided in Appendix 1.

Participants attended one physiotherapist-supervised clinic-based session and one physiotherapist-supervised online session via video conferencing (via WhatsApp video calls) each week, with each session lasting approximately 45–55 min. During the online sessions, PSSE was followed by conventional core stabilization and balance exercises. Participants also performed the prescribed home exercise program 3 days per week for approximately 45–55 min per session. Throughout the treatment period, participants in the CG were instructed to refrain from any additional exercise or rehabilitation intervention outside the study protocol, and their families were informed accordingly. Adherence to brace use and exercise participation was monitored using a Brace and Exercise Diary completed by the participants and verified by their families.

### Rehabilitative game-based exercise intervention

In the RGBEG, the initial level was completed using the same conventional core stabilization and balance exercises as in the CG. To prevent potential difficulties and between-group differences during the introduction and practice of transversus abdominis activation and neutral positioning of the spine and pelvis, the first 4 weeks of the program were administered using the same procedure in both groups. In the subsequent stages, core stabilization and balance exercises were performed using the Balance Maze and Balance Surf games in the BeCure Balance system in the clinical setting and the Smart Pose-based Runner and Gates games as part of the home program. The game-based tasks were progressively advanced by modifying speed, difficulty level, support-surface characteristics, and extremity involvement. In both groups, one clinic-based session and one online session (via WhatsApp video calls) were conducted each week, each lasting approximately 45–55 min; the home exercise program was performed 3 days per week for 45–55 min. Details of the exercise training are provided in Appendix 1. The therapeutic goals, game mechanics, feedback features, progression methods, and the physiotherapist's role for each game are presented in Appendix 2. Intervention-related adverse events were monitored and recorded by the research physiotherapist throughout the 24-week intervention period.

### Outcome measures

Assessments were performed at T0 (baseline), T1 (12 weeks), and T2 (24 weeks). Radiographic measurements (Cobb angle and Risser grade) were evaluated only at T0 and T2.

### Primary outcome measures

#### Cobb angle

Cobb angles were measured on standing posteroanterior radiographs using the standard Cobb method by a single physician who was blinded to group allocation [10]. Curves at all spinal levels were measured, and curve progression was defined as an increase of > 5° from baseline.

#### Posterior Trunk Symmetry Index (POTSI)

The POTSI is a photographic scoring system that quantifies posterior trunk asymmetry based on six indices derived from eight anatomical landmarks. Scores > 10 indicate impaired symmetry [11].

#### Walter Reed Visual Assessment Scale (WRVAS)

The WRVAS is a seven-item self-report scale scored from 7 to 35 that evaluates the participant's subjective perception of spinal deformity, with higher scores indicating greater perceived deformity [12].

### Secondary outcome measures

#### Angle of Trunk Rotation (ATR)

The angle of trunk rotation was measured in degrees at the thoracic, thoracolumbar, lumbar, and sacral levels using a scoliometer during Adam's forward bend test. Higher values indicate greater rotational asymmetry of the trunk [13]. Measurements were performed at T0, T1, and T2.

#### Physical Activity Participation Motivation Scale (MSPPA)

The MSPPA is a validated 16-item, 5-point Likert-type scale scored from 16 to 80 that measures motivation to participate in physical activity and comprises three subscales: individual reasons, environmental reasons, and amotivation [14].

#### Single-Leg Balance Test (SLBT)

The SLBT is a timed unilateral stance test performed bilaterally with the eyes open for 30 s. Failure criteria were the raised foot touching the stance limb, use of external support, the raised foot touching the floor, or hopping [15].

#### System Usability Scale (SUS)

The SUS is a 10-item, 5-point Likert-type scale that yields a score from 0 to 100; scores > 68 indicate above-average usability [16, 17, 18]. SUS assessments were performed only in the RGBEG. The first usability assessment was conducted after a 2-week familiarization period with the system. Subsequent assessments were repeated at weeks 12 and 24. This approach was selected to prevent the learning effect during the initial period of use from artificially lowering perceived usability.

### Statistical analysis

Statistical analyses were performed using IBM SPSS Statistics version 30.0 (IBM Corp., Armonk, NY, USA). Statistical significance was set at *p* < 0.05. The normality of continuous variables was assessed using the Shapiro–Wilk test. Normally distributed data were analyzed using parametric tests, whereas nonnormally distributed data were analyzed using nonparametric tests. Descriptive statistics are presented as mean ± standard deviation (SD) or median (interquartile range [IQR]), depending on the distribution of the data.

For within-group comparisons, the Wilcoxon signed-rank test was used for two repeated measurements, whereas the Friedman test was used for outcomes assessed at three time points.

For between-group comparisons, the independent-samples t-test or Mann–Whitney U test was used, as appropriate for the data distribution. Cohen's *d* was reported as the effect size for independent-samples *t*-tests, whereas rank-biserial correlation was reported for Mann–Whitney U tests. In addition, 95% confidence intervals were reported for all principal between-group comparisons. To adjust for potential baseline variability and obtain a more precise estimate of the intervention effect, additional ANCOVA models were performed for the MSPPA outcome. Separate ANCOVA models were conducted for the 12-week (T1) and 24-week (T2) assessments using baseline MSPPA (T0) as a covariate. The assumption of homogeneity of regression slopes was evaluated before the analyses. Partial eta squared (η^2^ₚ) was reported as the measure of effect size for ANCOVA.

Analyses were conducted according to a per-protocol approach and included participants who completed the 24-week intervention and follow-up assessments. Participants who did not demonstrate adequate adherence to the treatment protocol after randomization were not included in the final analyses. No imputation method was applied for missing data. A per-protocol approach was adopted because this pilot trial was primarily designed to assess feasibility and preliminary efficacy signals under conditions of adequate treatment exposure. However, this approach may overestimate treatment efficacy, as participants with poor adherence or incomplete follow-up were excluded, and it may limit the generalizability of the findings to the broader population of patients receiving this intervention, including those with variable adherence.

## Results

### Demographic and clinical characteristics

A total of 25 female patients with AIS were randomized. After randomization, 12 participants were allocated to the CG and 13 to the RGBEG. During the 24-week intervention, 2 participants in the CG were lost to follow-up because of insufficient compliance with brace and exercise treatment. In the RGBEG, 3 participants were lost to follow-up because of personal reasons and insufficient brace use. The final analyses included 20 participants, with 10 participants in each group. Participant flow is presented in the CONSORT flow diagram (Fig. 1).

There were no statistically significant between-group differences in demographic or clinical characteristics at baseline (*p* > 0.05; Table 1). No intervention-related adverse events were observed in either group during the 24-week intervention period.

**Table 1:** Baseline demographic and clinical characteristics of the participants

	**Control Group (*n* = 10)**		**RGBEG (*n* = 10)**		**p**	**95% CI**	**Effect Size**
	**Mean ± SD (min–max.)**	**Median (IQR)**	**Mean ± SD (min–max.)**	**Median (IQR)**			
**Age (m)**	140.50 ± 14.82 (125–172)	138 (22)	140.40 ± 12.91 (120–156)	142.50 (25)	0.98^b^	(−12.96, 13.16)	0.01
**Height (cm)**	150.00 ± 8.14 (134–164)	149.50 (10)	154.20 ± 8.00 (145–168)	153.50 (14)	0.26^b^	(−11.78,3.38)	0.52
**Weight (kg)**	42.20 ± 8.42 (30–61)	41.50 (9)	45.70 ± 7.30 (38–58)	45.00 (13)	0.33^b^	(−10.90, 3.90)	0.44
**BMI (kg/m**^**2**^**)**	18.57 ± 1.85 (16.67–22.68)	18.05 (2.84)	19.21 ± 2.72 (15.42–24.14)	18.29 (3.16)	0.54^b^	(−2.83, 1.54)	0.27
**Menarche (m)**	3.20 ± 4.13 (0–10)	0.50 (8)	2.80 ± 4.37 (0–12)	0.00 (5)	0.74^a^	(−3.00,5.00)	0.09
**Thoracic Cobb°**	18.50 ± 16.16 (0–36)	27.00 (33)	23.70 ± 10.61 (0–40)	25.00 (10)	1.00^a^	(−25.00, 8.00)	0.00^e^
**Lumbar Cobb°**	16.90 ± 12.62 (0–33)	19.00 (27)	23.20 ± 10.42 (0–37)	26.00 (10)	0.24^b^	(−17.17, 4.57)	0.54
**Thoracolumbar Cobb°**	9.00 ± 14.77 (0–37)	0.00 (26)	3.30 ± 10.44 (0–33)	0.00 (0)	0.33^a^	(0.00, 26.00)	0.19
**Thoracic ATR**	7.20 ± 3.88 (3–14)	6.00 (7)	7.20 ± 3.01 (4–14)	7.50 (4)	1.00^b^	(−3.26, 3.26)	0.00^e^
**Lumbar ATR**	5.10 ± 2.42 (3–10)	4.50 (4)	5.10 ± 1.52 (3–7)	4.50 (3)	1.00^b^	(−1.90, 1.90)	0.00^e^
**Thoracolumbar ATR**	5.10 ± 2.38 (1–10)	4.50 (2)	4.90 ± 1.60 (3–8)	5.00 (2)	0.83^b^	(−1.70, 2.10)	0.01
**Risser Stage**	0.40 ± 0.52 (0–1)	0.00 (1)	0.30 ± 0.68 (0–2)	0.00 (0)	0.48^a^	(0.00, 1.00)	0.16

^a^Mann-Whitney U test; *p* < 0.05

^b^Independent-samples t-test; *p* < 0.05

CI: Confidence interval

^e^Rank-biserial correlation for Mann–Whitney U test

*ATR* Angle of Trunk Rotation, *BMI* Body Mass Index, *CG* Control Group, *RGBEG* Rehabilitative Game-Based Exercise Group

### Cobb angle

Within-group analysis showed a statistically significant reduction in thoracic Cobb angle from T0 to T2 in both the CG (*p* = 0.03) and RGBEG (*p* = 0.03). A significant within-group change in lumbar Cobb angle was observed only in the RGBEG (*p* = 0.04), whereas the change in the CG did not reach statistical significance (*p* = 0.45). No significant change was detected at the thoracolumbar level in either group (Table 2).

**Table 2:** Within-group and between-group comparisons of Cobb Angles

**Within Group**				**Between Groups**						
		**CG** **p**	**RGBEG** **p**	**CG**		**RGBEG**		**p**	**95%** **CI**	**Effect Size**
				**Mean ± SD**	**Median (IQR)**	**Mean ± SD**	**Median (IQR)**			
**Cobb°**	T (T0 – T2)	**0.03**^**c**^	**0.03**^**c**^	−2.90 ± 2.89	−3.00 (5)	−2.00 ± 2.45	−3.00 (5)	0.46^b^	(−3.41, 1.61)	0.34
	TL (T0 – T2)	0.11^c^	0.32^c^	−1.30 ± 2.21	0.00 (3.25)	−0.30 ± 0.95	0.00 (0)	0.26^a^	(−3.00,0.00)	0.22
	L (T0 – T2)	0.45^c^	**0.04**^**c**^	−1.20 ± 4.87	−0.50 (6)	−2.20 ± 2.90	−2.50 (4)	0.58^b^	(−2.77, 4.77)	0.25

^a^Mann-Whitney U test; *p* < 0.05

^b^Independent-samples t-test; *p* < 0.05

^c^Wilcoxon signed-rank test; *p* < 0.05

*CI* Confidence interval, *CG* Control Group, *RGBEG* Rehabilitative Game-Based Exercise Group

Between-group comparisons showed no significant differences in T0–T2 change scores for the thoracic, lumbar, or thoracolumbar Cobb angles (*p* = 0.46, *p* = 0.58 and *p* = 0.26, respectively). Within- and between-group comparisons of Cobb angles are presented in Table 2.

### Angle of trunk rotation

Within-group comparisons showed statistically significant reductions from T0 to T2 in thoracic, thoracolumbar, and lumbar ATR in both groups (CG: *p* = 0.006, *p* = 0.003, and *p* < 0.001, respectively; RGBEG: *p* < 0.001, *p* = 0.03, and *p* = 0.004, respectively). No significant change was observed at the sacral level in either group. There were no statistically significant between-group differences in T0–T2 change scores at any spinal level (*p* > 0.05).

### Posterior trunk symmetry index

Within-group comparisons showed a statistically significant change in POTSI scores over the treatment period in both groups (CG: *p* = 0.005; RGBEG: *p* = 0.005).

Between-group comparisons of POTSI scores showed no statistically significant difference in T0–T2 change scores (*p* = 0.80). Between-group comparisons of POTSI scores from T0 to T2 are presented in Table 3.

**Table 3:** Within-group and between-group comparisons of T0–T2 change scores for POTSI and WRVAS

Within Group			Between Groups						
**Parameter**	CG p	RGBEG p	CG		RGBEG		p	95% CI	Effect Size
			**Mean ± SD**	**Median (IQR)**	**Mean ± SD**	**Median (IQR)**			
**POTSI T0–T2**	**0.005**^**c**^	**0.005**^**c**^	−4.51 ± 1.66	−5.24 (2.48)	−4.77 ± 2.55	−4.58 (3.08)	0.80ᵇ	(−1.77, 2.27)	0.12
**WRVAS T0–T2**	**0.005**^**c**^	**0.005**^**c**^	−4.20 ± 1.62	−4.00 (2)	−5.40 ± 1.84	−5.00 (3)	0.14ᵇ	(−0.43, 2.83)	0.69

*POTSI* Posterior Trunk Symmetry Index, *WRVAS* Walter Reed Visual Assessment Scale, *CG* Control Group, *RGBEG* Rehabilitative Game-Based Exercise Group, *CI* Confidence interval, *T0* Baseline, *T2* Month 6. The T0–T2 row represents the change score

^b^Independent-samples t-test; *p* < 0.05

^c^Wilcoxon signed-rank test; *p* < 0.05

### Walter reed visual assessment scale

Within-group comparisons showed a significant change in WRVAS scores over the treatment period in both groups (CG: *p* = 0.005; RGBEG: *p* = 0.005).

Between-group comparisons of WRVAS scores showed no statistically significant difference in T0–T2 change scores (*p* = 0.14). Between-group comparisons of WRVAS scores from T0 to T2 are presented in Table 3.

### Motivation to participate in physical activity

At baseline, no significant difference in total MSPPA scores was observed between the CG and the RGBEG (*p* = 0.12). In the within-group analysis, MSPPA scores decreased significantly from T0 to T2 in the CG (*p* = 0.005), whereas no significant change was observed in the RGBEG (*p* = 0.65). Although no significant between-group differences were observed in the absolute MSPPA scores at T0, T1, or T2 (*p* > 0.05), between-group comparisons of the change scores demonstrated a significantly smaller decline in motivation in the RGBEG than in the CG at both T0–T1 (*p* = 0.02) and T0–T2 (*p* < 0.001) (Table 4).

**Table 4:** Within-group and between-group comparisons of the physical activity participation motivation scale

	**Within Group**		**Between Groups**							
**MSPPA**	**CG** **p**	**RGBEG** **p**		**CG**		**RGBEG**		**p**	**95% CI**	**Effect Size**
				**Mean ± SD**	**Median (IQR)**	**Mean ± SD**	**Median (IQR)**			
T0-T1-T2	**0.004**^**d**^	0.83^d^	T0	33.70 ± 7.90	33.00 (10)	27.84 ± 2.32	29.00 (4)	0.12^b^	(−1.27, 9.67)	0.72
T0-T1	**0.01**^**c**^	0.92^c^	T1	26.60 ± 7.10	25.00 (11)	27.75 ± 2.60	30.00 (4)	0.22^b^	(−8.00,2.00)	0.56
T0-T2	**0.005**^**c**^	0.65^c^	T2	25.00 ± 8.14	24.00 (13)	29.60 ± 3.98	29.50 (6)	0.13^b^	(−10.62, 1.42)	0.72
			(T0-T1)	−7.10 ± 8.61	−6.00 (6)	0.10 ± 2.51	0.00 (4)	**0.02**^**b**^	(−13.16, −1.24)	1.14
			(T0-T2)	−8.70 ± 7.12	−7.00 (4)	0.10 ± 4.12	−1.50 (5)	** < 0.001**^**a**^	(−11.00,−4.00)	0.94

^a^Mann–Whitney U test; *p* < 0.05

^b^Independent-samples t-test; *p* < 0.05

^c^Wilcoxon signed-rank test; *p* < 0.05

^d^Friedman test

*CI* Confidence interval, *T0* Baseline, *T2* Month 6. The T0–T2 row represents the change score

*MSPPA* Physical Activity Participation Motivation Scale

To further evaluate the intervention effect and adjust for potential baseline variability in physical activity motivation, additional ANCOVA analyses were performed using baseline MSPPA (T0) as a covariate. The assumption of homogeneity of regression slopes was satisfied in both models (GROUP × baseline interaction, *p* > 0.05). After adjustment for baseline MSPPA, no significant between-group difference was observed at T1 (F(1,17) = 1.72, *p* = 0.207, partial η^2^ = 0.092). However, the between-group difference at T2 remained statistically significant (F(1,17) = 7.37, *p* = 0.015, partial η^2^ = 0.303), indicating that the between-group difference at 24 weeks remained significant after adjustment for baseline MSPPA.

### Balance performance

For the SLBT performed on the right and left sides, within-group comparisons showed no significant change from T0 to T2 in either group (CG right: *p* = 0.07; CG left: *p* = 0.11; RGBEG right: *p* = 0.11; RGBEG left: *p* = 0.32). Between-group comparisons also showed no significant differences in T0–T2 change scores for either side (right: *p* = 0.53; left: *p* = 0.48) (Table 5).

**Table 5:** Within-group and between-group comparisons of T0–T2 changes in right and left single-leg balance performance

Within Group			Between Groups						
	CG p	RGBEG p	CG		RGBEG		p	95% CI	Effect Size
			**Mean ± SD**	**Median (IQR)**	**Mean ± SD**	**Median (IQR)**			
**Right L** **T0–T2**	0.07^c^	0.11^c^	3.00 ± 4.76	0.00 (2.25)	0.90 ± 1.73	0.00 (1.50)	0.53^a^	(0.00, 5.00)	0.18
**Left L T0–T2**	0.11^c^	0.32^c^	1.50 ± 3.17	0.00 (2.25)	0.30 ± 0.95	0.00 (0)	0.48^a^	(0.00, 2.00)	0.20

^a^Mann–Whitney U test; *p* < 0.05

^c^Wilcoxon signed-rank test; *p* < 0.05

*CI* Confidence interval, *CG* Control Group, *RGBEG* Rehabilitative Game-Based Exercise Group, *L* Leg

### System usability

The usability of the rehabilitative game-based system was evaluated using the SUS only in the RGBEG. Because the first assessment (T0) was obtained after a 2-week familiarization period, it was not concurrent with T0 assessments of the other outcome measures. System usability remained above the acceptable usability threshold of 68 at all assessment time points, with a median score of 76.50. The maintenance of usability throughout the treatment period indicates that the system was considered acceptable not only during initial use but also during long-term use (Table 6).

**Table 6:** Comparison of total system usability scale scores across assessment time points

Parameter	T0 Median (IQR)	T1 Median (IQR)	T2 Median (IQR)	p (T0-T1-T2)	p (T0-T1)	p (T1-T2)	p (T0-T2)
Total Score	76.50 (20)	76.50 (10)	76.50 (18)	0.43^d^	0.63^c^	0.68^c^	0.72^c^

^c^Wilcoxon signed-rank test; *p* < 0.05

^d^Friedman test

## Discussion

In nonoperative treatment of AIS, inadequate adherence is associated with suboptimal clinical outcomes. With respect to exercise, adherence to PSSE programs has been reported to decline over time, and when motivation is insufficient, patients may fail to perform the prescribed frequency, intensity, or duration of the exercise intervention [18]. Therefore, when evaluating the clinical effectiveness of exercise-based interventions, attention should be given not only to the exercise content but also to strategies that support sustained patient engagement in the program. In this context, technology-based game approaches were used in our study as a motivating treatment component, and their effects were examined using clinical and radiological parameters.

Regarding the primary radiological outcome, no additional advantage of the rehabilitative game-based exercise approach over the conventional exercise approach was detected in terms of Cobb angle. However, both groups showed significant reductions in thoracic Cobb angle over time. Consistent with our findings, Yuan et al. [19] investigated the effectiveness of individualized PSSE delivered through a digital platform in patients with AIS and reported improvements in Cobb angle in both the digital and conventional face-to-face groups, although the improvement was more pronounced in the digital care group. The discrepancy between that finding and our results may be related to the limited sample size inherent in our pilot design, which reduced the statistical power to detect between-group differences. In one of the earliest studies of rehabilitative game systems, Wibmer et al. [20] reported no progression in Cobb angle after 6 months among patients with AIS who performed game-assisted Schroth exercises. The favorable radiological course observed in our study suggests that the feedback mechanisms provided by digital systems in both clinical and home settings may be relevant to supporting exercise quality and continuity. A recent systematic review reported that hybrid models combining web-based and face-to-face approaches may more effectively meet patient needs and thereby improve both the effectiveness and sustainability of rehabilitation [19]. This finding supports our clinical inference that game-based digital interventions may be considered a complementary option to conventional approaches in AIS rehabilitation. However, the absence of significant between-group differences should not be interpreted as evidence of equivalence, as this pilot trial was not designed for equivalence or non-inferiority testing.

Given the small per-protocol sample, the non-significant between-group differences observed for Cobb angle and other clinical outcomes may reflect limited statistical power rather than true equivalence between the two treatment approaches. Future trials should therefore be adequately powered based on effect sizes derived from this pilot study and should employ an intention-to-treat analysis to provide more robust and generalizable estimates of efficacy.

The other primary outcomes addressed cosmetic appearance, an important treatment goal in AIS [1], by evaluating trunk symmetry objectively using POTSI and patients’ subjective perception of body appearance using WRVAS. Both POTSI and WRVAS scores improved significantly in both groups compared with baseline. Although studies examining the effects of technology-based systems on body appearance remain limited, the available evidence highlights their potential in this area. Yuan et al. [19] reported significant improvements in WRVAS scores with an individualized exercise program delivered through a digital platform, whereas Takinacı et al. [21] reported significant improvements in postural alignment and functional outcomes in patients with AIS who received telerehabilitation support in addition to an artificial intelligence-assisted home exercise program. The systematic review by Li et al. [8] emphasized that digital applications may support engagement in rehabilitation, and consequently treatment outcomes, through feedback, interaction, and remote monitoring features. In our study, no statistically significant between-group differences were detected in POTSI and WRVAS change scores. Together with the significant improvements observed over time, these findings suggest that game-based digital exercise has potential as a complementary component of conservative AIS treatment.

Regarding the secondary balance outcome, SLBT performance did not change significantly from baseline to the 6-month follow-up in either group, and no significant between-group difference was detected. This finding is consistent with some reports in the literature. Meng [22], Xu [23] and Piątek-Krzywicka et al. [24] reported that balance parameters were not markedly impaired in individuals with AIS and that the balance function required for activities of daily living was generally preserved. In contrast, Xuan et al. [25] and Şahin et al. [26] reported reduced static balance ability. The inconsistent findings regarding balance function in individuals with scoliosis warrant cautious interpretation of our result. Several factors may explain the absence of a significant change in balance in our study. First, because our sample consisted of patients with mild-to-moderate curves, balance impairment may not yet have been clinically apparent. In addition, preservation of visual input during the SLBT and the absence of increased proprioceptive or vestibular challenge may have limited the test's ability to identify balance-related impairment. Li et al. [27] reported that balance function in individuals with AIS may be influenced by several factors, including the testing method, measurement equipment, scoliosis severity, age, skeletal maturity, and muscle strength. Therefore, future studies should comprehensively assess balance function in individuals with AIS while accounting for these factors to improve the reliability of the findings.

Among the secondary outcomes, the most distinctive finding was that motivation to participate in physical activity declined in the CG but was maintained in the RGBEG over the 6-month follow-up. Importantly, the between-group difference at 24 weeks remained statistically significant after adjustment for baseline motivation. The prolonged and repetitive nature of nonoperative treatments may make it difficult to maintain motivation and treatment engagement, particularly among adolescents [28]. The literature suggests that gamification elements may support motivational change. A review of individuals with AIS reported that, in the absence of an external support mechanism, patients had difficulty maintaining exercise programs and implementing the behavioral changes required for treatment adherence [4]. In this context, our findings suggest that rehabilitative game systems may provide external motivational support during the behavioral change process. However, a study of individuals with AIS performing exergame-based exercise at home reported that interest in the games and exercise frequency declined over time [20]. The inability to digitize more than two exercises and the use of a fixed difficulty level throughout treatment were suggested as possible explanations. The absence of a similar motivational decline in our study may be related to the varied game content, progressive task structure, and diverse visual stimuli. These features may have contributed to maintaining motivation, however, whether this motivational pattern translated into greater treatment adherence remains uncertain because adherence was not objectively quantified using comparable measures in both groups. Therefore, the motivation finding should be considered a promising but preliminary signal that requires confirmation using objective adherence measures in future trials.

Evaluating how users perceive game-based exercise systems, a relatively new application in AIS rehabilitation, is important for determining their clinical feasibility. In our study, system usability assessed using the SUS fell within the 'good usability' category accepted in the literature at all assessment time points [29]. Importantly, usability was maintained throughout the treatment period and was not limited to the initial assessment. These findings are consistent with studies using similar methodological designs. Kamnardsiri et al. [30] reported good-to-excellent SUS scores for a gamified exercise system delivered via a computer camera in older adults, whereas Zhao et al. [31] reported that a simple design positively influenced user engagement and perceived usability in a monocular camera-based home exercise system. In our study, the ability to use the home-based Smart Pose component with only a phone or tablet equipped with a front-facing camera, its compatibility with different mobile devices, simple login requirements, and exercise content aligned with therapeutic goals may explain the high usability scores. Savage et al. [32] reported that multi-device compatibility may improve perceived usability and acceptability in digital health applications, which is consistent with the design choices in our study.

## Conclusion

This pilot randomized controlled trial evaluated the clinical feasibility and preliminary effects of rehabilitative game-based exercise integrated into conservative treatment in skeletally immature female patients with AIS. Both groups showed significant improvements over time in thoracic Cobb angle, trunk symmetry, and perceived body appearance, while no statistically significant between-group differences were detected in these outcomes. The most notable finding was that physical activity motivation declined significantly in the CG during the 6-month follow-up, whereas motivation scores were maintained in the RGBEG. This result suggests that rehabilitative game systems may represent a feasible and acceptable complementary approach that may support sustained motivation during long-term conservative AIS rehabilitation. Because of the pilot design and limited sample size, the findings should be interpreted as exploratory and confirmed in larger studies.

### Limitations

This study has several limitations. First, because the study targeted skeletally immature individuals with AIS, brace use—an integral component of conservative treatment—may have substantially influenced several outcomes, particularly Cobb angle and body appearance, making it difficult to attribute the findings solely to the game-based system. Second, because of the three-dimensional and complex nature of PSSE, not all exercises could be fully implemented through the digital platforms. Third, owing to the nature of exercise interventions, complete blinding of participants and therapists to group allocation was not possible. This may have increased the risk of performance bias. In addition, because participants' awareness of group allocation may have influenced responses to self-reported measures such as the WRVAS, MSPPA, and SUS, these findings should be interpreted cautiously. Exercise participation was monitored using diaries verified by participants’ families, and completion of home exercises in the RGBEG was verified using digital usage records. However, comparable objective adherence measures were not available for both groups. Therefore, the maintenance of motivation in the RGBEG should not be interpreted as direct evidence of improved treatment adherence. Future studies should assess objective adherence measures, such as exercise duration, completed sessions, and digital usage records, in both groups. Finally, the pilot nature of the study limits the generalizability of the findings, which should be confirmed in larger samples and multicenter studies.

## Supplementary Information

Supplementary Material 1.

Supplementary Material 2.

Supplementary Material 3.

## Data Availability

The datasets generated and/or analyzed during the current study are available from the corresponding author upon reasonable request.

## Abbreviations

AIS: Adolescent Idiopathic Scoliosis
ATR: Angle of Trunk Rotation
CG: Control Group
RGBEG: Rehabilitative Game-Based Exercise Group
SLBT: Single Leg Balance Test
SD: Standard Deviation
WRVAS: Walter Reed Visual Assessment Scale
POTSI: Posterior Trunk Symmetry Index
SUS: System Usability Scale
PSSE: Physiotherapeutic Scoliosis-Specific Exercises
BSPTS: Barcelona Scoliosis Physical Therapy School
MSPPA: Physical Activity Participation Motivation Scale

## References

[1] Negrini S, Donzelli S, Aulisa AG, Czaprowski D, Schreiber S, de Mauroy JC, et al. 2016 SOSORT guidelines: orthopaedic and rehabilitation treatment of idiopathic scoliosis during growth. Scoliosis Spinal Disord. 2018;13:3. 10.1186/s13013-017-0145-8 PMC5795289 29435499

[2] Koura G, Elshiwi AMF, Reddy RS, Alrawaili SM, Ali ZA, Alshahrani MAN, et al. Proprioceptive deficits and postural instability in adolescent idiopathic scoliosis: a comparative study of balance control and key predictors. Front Pediatr. 2025;13:1595125. 10.3389/fped.2025.1595125 PMC12198138 40574958

[3] Kuang H, Chen L, Huang M, Chen J. Management of adolescent scoliosis: a comprehensive review of etiology and rehabilitation. Front Pediatr. 2025;13:1596400. 10.3389/fped.2025.1596400 PMC12307321 40740816

[4] Fazalbhoy A, McAviney J, Mirenzi R. Compliance of physiotherapeutic scoliosis-specific exercise in adolescent idiopathic scoliosis: a scoping review. J Clin Med. 2025;14(9):2950. 10.3390/jcm14092950 PMC12072716 40363981

[5] Sykorova K, Mathew A, Pavel N, Gazerani P, Saidi T, Johnsen MB, et al. Exploring stakeholders’ perceptions of using digital health technologies to improve the conservative treatment of adolescent idiopathic scoliosis: qualitative study. J Med Internet Res. 2025;27:e69089. 10.2196/69089 PMC12242061 40561463

[6] Wei W, Tang H, Luo Y, Yan S, Ji Q, Liu Z, et al. Efficacy of virtual reality exercise in knee osteoarthritis rehabilitation: a systematic review and meta-analysis. Front Physiol. 2024;15:1424815. 10.3389/fphys.2024.1424815 PMC11220424 38962070

[7] Merola P, Cardoso MB, Barreto G, Chagas MC, Farias Oliveira Saunders L, Saunders B, et al. Virtual reality high-intensity interval training exergaming compared to traditional high-intensity circuit training among medical students: pilot crossover study. JMIR Serious Games. 2025;13:e63461. 10.2196/63461 PMC11731695 39773879

[8] Li Y, Chang F, Zhang W, Ren Z, Chen Y, Liu Z. Behavior change strategies in digital exercise interventions for adolescent idiopathic scoliosis: scoping review. J Med Internet Res. 2025;27:e66981. 10.2196/66981 PMC12485258 40957016

[9] Gür G, Ayhan C, Yakut Y. The effectiveness of core stabilization exercise in adolescent idiopathic scoliosis: a randomized controlled trial. Prosthet Orthot Int. 2017;41(3):303–10. 10.1177/0309364616664151 27625122

[10] Kelly J, Freetly T, Dekis J, et al. Treatment of adolescent idiopathic scoliosis and evaluation of the adolescent patient. Curr Orthop Pract. 2018;29(1):1–10.

[11] El-Hawary R, Chukwunyerenwa C. Update on evaluation and treatment of scoliosis. Pediatr Clin North Am. 2014;61(6):1223–41. 10.1016/j.pcl.2014.08.007 25439021

[12] Pineda S, Bago J, Gilperez C, Climent JM. Validity of the Walter Reed Visual Assessment Scale to measure subjective perception of spine deformity in patients with idiopathic scoliosis. Scoliosis. 2006;1:18. 10.1186/1748-7161-1-18 PMC1654183 17090338

[13] Bunnell WP. An objective criterion for scoliosis screening. J Bone Joint Surg Am. 1984;66(9):1381–7. 6501335

[14] Tekkursun Demir G, Cicioğlu I. Physical Activity Participation Motivation Scale (PAPMS): a validity and reliability study. J Hum Sci. 2018;15(4):2479.

[15] Springer BA, Marin R, Cyhan T, Roberts H, Gill NW. Normative values for the unipedal stance test with eyes open and closed. J Geriatr Phys Ther. 2007;30(1):8–15. 10.1519/00139143-200704000-00003 19839175

[16] Brooke J. SUS: a “quick and dirty” usability scale. In: Jordan PW, Thomas B, Weerdmeester BA, McClelland IL, editors. Usability evaluation in industry. London: Taylor & Francis; 1996. p. 189–94.

[17] Çağıltay K. Human-computer interaction and usability engineering: from theory to practice. 1st ed. Ankara: METU Development Foundation Publishing; 2011.

[18] Simhon ME, Fields MW, Grimes KE, Bakarania P, Matsumoto H, Boby AZ, et al. Completion of a formal physiotherapeutic scoliosis-specific exercise training program for adolescent idiopathic scoliosis increases patient compliance to home exercise programs. Spine Deform. 2021;9(3):785–92. 10.1007/s43390-020-00253-8 33230667

[19] Yuan W, Shi W, Chen L, Liu D, Lin Y, Li Q, et al. Digital physiotherapeutic scoliosis-specific exercises for adolescent idiopathic scoliosis: a randomized clinical trial. JAMA Netw Open. 2025;8(2):e2459929. 10.1001/jamanetworkopen.2024.59929 PMC11836762 39964686

[20] Wibmer C, Groebl P, Nischelwitzer A, Salchinger B, Sperl M, Wegmann H, et al. Video-game-assisted physiotherapeutic scoliosis-specific exercises for idiopathic scoliosis: case series and introduction of a new tool to increase motivation and precision of exercise performance. Scoliosis Spinal Disord. 2016;11:44. 10.1186/s13013-016-0104-9 PMC5121954 27896317

[21] Takinaci ZD, Çelik M, Yildiz Ş, Talmac MA, Dut R. Enhancing home-based exercise therapy with telerehabilitation in mild adolescent idiopathic scoliosis: a randomized controlled trial. Healthcare. 2026;14(1):19. 10.3390/healthcare14010019 PMC12785525 41516952

[22] Meng FY, Zhao YX, Yuan Y, et al. Static stability and stability limit for patients with scoliosis. Chin J Rehabil Theory Pract. 2021;27:1351–5.

[23] Xu JL. Functional assessment of postural balance in patients with idiopathic scoliosis. Wuhan: Wuhan Sports University; 2022.

[24] Piatek-Krzywicka E, Borzucka D, Kuczynski M. Postural control through force plate measurements in female AIS patients compared to their able-bodied peers. Sci Rep. 2022;12(1):13170. 10.1038/s41598-022-17597-y PMC9343654 35915125

[25] Xuan Z, Qing D, Li Z, et al. Study of static balance in patients with adolescent idiopathic scoliosis. Chin J Rehabil Med. 2010;25:953–6.

[26] Şahin F, Urak O, Akkaya N. Evaluation of balance in young adults with idiopathic scoliosis. Turk J Phys Med Rehabil. 2019;65:236–43. 10.5606/tftrd.2019.2825 PMC6797924 31663072

[27] Li SH, Wang LC, Shen HJ. A study on static balance ability in 48 patients with adolescent idiopathic scoliosis. Med Recapitulate. 2019;25:2897–902.

[28] Chicca J, Shellenbarger T. Generation Z: approaches and teaching-learning practices for nursing professional development practitioners. J Nurses Prof Dev. 2018;34(5):250–6. 10.1097/NND.0000000000000478 30188477

[29] Zanatta F, Giardini A, Pierobon A, D’Addario M, Steca P. A systematic review on the usability of robotic and virtual reality devices in neuromotor rehabilitation: patients’ and healthcare professionals’ perspective. BMC Health Serv Res. 2022;22(1):523. 10.1186/s12913-022-07821-w PMC9020115 35443710

[30] Kamnardsiri T, Kumfu S, Munkhetvit P, Boripuntakul S, Sungkarat S. Home-based, low-intensity, gamification-based interactive physical-cognitive training for older adults using the ADDIE model: design, development, and evaluation of user experience. JMIR Serious Games. 2024;12:e59141. 10.2196/59141 PMC11536494 39470391

[31] Zhao J, Zhang Y, Liu Y, Chen X, Li J. Development and evaluation of a monocular camera-based mobile exergame for at-home intervention in individuals at high risk of type 2 diabetes: a randomized controlled trial. JMIR Serious Games. 2025;13:e55273. 10.2196/75823 PMC12699957 41384807

[32] Savage LC, Soto-Cossio LE, Minardi F, et al. The development of a digital patient navigation tool to increase colorectal cancer screening among federally qualified health center patients: acceptability and usability testing. JMIR Form Res. 2024;8:e53224. 10.2196/53224 PMC11464930 39321451

