# Supplementary material for: Rehabilitative game-based exercise integrated into conservative treatment for adolescent idiopathic scoliosis: a pilot randomized controlled trial
Source: BMC Sports Sci Med Rehabil. 2026 Aug 18;18:590. doi: 10.1186/s13102-026-01981-6 (PMC13761108; doi:10.1186/s13102-026-01981-6)
Supplement: Supplementary file 1 [file 13102_2026_1981_MOESM1_ESM.docx]

**Table 1. Comparison of Intervention Protocols**

| **Component** | **Control Group (CG)** | **RGBEG** |
| --- | --- | --- |
| **Bracing** | Three-dimensional rigid Chêneau-type brace, full-time (≥20 hours/day) | Three-dimensional rigid Chêneau-type brace, full-time (≥20 hours/day) |
| **PSSE (BSPTS)** | 3 levels × 8 weeks; 3×6 repetitions in weeks 1–4, 4×8 repetitions in weeks 5–8; individualized according to curve type | 3 levels × 8 weeks; 3×6 repetitions in weeks 1–4, 4×8 repetitions in weeks 5–8; individualized according to curve type |
| **Core stabilization** | Conventional method: transversus abdominis (TrA) activation → bridge exercises → resistance-based stabilization; progressive | Introductory level identical to CG protocol; thereafter integrated with BeCure Balance system in clinic (Balance Maze and Balance Surf games) |
| **Balance training** | Conventional method: 5-level progressive program from eyes-open stance on firm surface to eyes-closed dynamic tasks on compliant surface (tandem stance, mini squat, lunge) | Smart Pose–based Runner and Gates games; applied on firm/compliant surfaces in tandem, squat, and lunge positions; progression via speed, difficulty level, surface type, and limb involvement |
| **Clinical session** | Once per week, supervised by physiotherapist, 45–55 min | Once per week, supervised by physiotherapist, 45–55 min (BeCure Balance system) |
| **Online session** | Once per week via video conferencing, physiotherapist-supervised, 45–55 min; PSSE followed by conventional core stabilization and balance exercises. | Once per week via video conferencing, physiotherapist-supervised, 45–55 min; PSSE followed by game-based core stabilization and balance exercises. |
| **Home program** | 3 days per week, 45–55 min; adherence monitored via exercise diary | 3 days per week, 45–55 min; Runner and Gates games; adherence monitored using exercise diaries and additionally verified through the BeCure Virtual Clinic System. |
| **Intervention duration** | 24 weeks (T0: baseline; T1: week 12; T2: week 24) | 24 weeks (T0: baseline; T1: week 12; T2: week 24) |

*PSSE: Physiotherapeutic Scoliosis-Specific Exercise; BSPTS: Barcelona Scoliosis Physical Therapy School; TrA: Transversus Abdominis; CG: Control Group; ROTEG: Rehabilitative Game-Based Exercise Group; T0: baseline; T1: 12th week; T2: 24th week.*

** The planned exercise frequency and session duration were matched between groups; the groups differed only in the delivery of core stabilization and balance exercises.*
