# Supplementary material for: Rehabilitative game-based exercise integrated into conservative treatment for adolescent idiopathic scoliosis: a pilot randomized controlled trial
Source: BMC Sports Sci Med Rehabil. 2026 Aug 18;18:590. doi: 10.1186/s13102-026-01981-6 (PMC13761108; doi:10.1186/s13102-026-01981-6)
Supplement: Supplementary file 2 [file 13102_2026_1981_MOESM2_ESM.docx]

**Table 1. Therapeutic and Game Design Characteristics of the Rehabilitative Game-Based Intervention**

| **Game** | **Setting and system** | **Player movement and game task** | **Therapeutic targets** | **Feedback and reward** | **Progression** | **Physiotherapist’s role** |
| --- | --- | --- | --- | --- | --- | --- |
| **Balance Maze** | Clinical setting; BeCure Balance system using a balance board and laptop camera | Participants shifted their body weight while maintaining the prescribed posture to move the on-screen balance board and guide a ball toward the target locations | Core stabilization, postural balance, controlled weight shifting, and visual-motor coordination | Real-time visual feedback, target achievement, score, and task duration | Game level and speed were adjusted weekly. Exercise difficulty was further increased by changing squat depth, upper-extremity position, external load, and task duration | Selected and manually adjusted the game level; monitored postural correction, trunk stability, movement quality, and safety |
| **Balance Surf** | Clinical setting; BeCure Balance system using a balance board and laptop camera | Participants controlled an on-screen surfboard through weight shifting and postural adjustments to reach reward zones on a moving water surface | Dynamic balance, trunk stabilization, multidirectional weight shifting, and visual-motor coordination | Real-time visual feedback, reward zones, score, and task duration | Game level and speed were adjusted weekly. Progression included changes in squat position, upper-extremity position, external load, and duration | Selected and manually adjusted the game level; supervised alignment, postural corrections, trunk control, and safety |
| **Runner** | Home and online sessions; BeCure Smart Pose application using the front camera of a smartphone or tablet | Participants moved an on-screen avatar using predefined unilateral or bilateral upper-extremity and body positions to avoid obstacles and increase their score | Core stabilization, postural control, upper-extremity integration, dynamic balance, and motor planning | Real-time visual avatar response, successful obstacle avoidance, and score | Progression was achieved by manually increasing game speed and difficulty and by changing the support surface, stance position, limb involvement, external load, and exercise duration | Prescribed the game and difficulty level through the BeCure Virtual Clinic System; monitored completion records and supervised movement accuracy during online sessions |
| **Gates** | Home and online sessions; BeCure Smart Pose application using the front camera of a smartphone or tablet | Participants positioned their body and extremities to move an on-screen avatar through approaching gates at the correct time and position | Multidirectional postural control, core stabilization, upper-extremity integration, balance, and motor planning | Real-time visual feedback, musical cues, successful gate passage, and score | Progression was achieved by manually increasing game speed and difficulty and by modifying the support surface, stance position, limb involvement, external load, and exercise duration | Prescribed the game and difficulty level through the BeCure Virtual Clinic System; monitored exercise completion and corrected movement quality during online sessions |

**Note.** *The first 4 weeks were identical in both groups and did not include game-based exercises. The game-based intervention was implemented from weeks 5 to 24. Game difficulty and progression were manually determined by the physiotherapist according to the participant’s performance. No intervention-related adverse events were observed.*
