# Supplementary material for: Rehabilitative game-based exercise integrated into conservative treatment for adolescent idiopathic scoliosis: a pilot randomized controlled trial
Source: BMC Sports Sci Med Rehabil. 2026 Aug 18;18:590. doi: 10.1186/s13102-026-01981-6 (PMC13761108; doi:10.1186/s13102-026-01981-6)
Supplement: Supplementary file 3 [file 13102_2026_1981_MOESM3_ESM.docx]

**CONSORT 2010 Checklist**

*Checklist of information to include when reporting a randomised trial*

**Manuscript:** Rehabilitative Game-Based Exercise Integrated into Conservative Treatment for Adolescent Idiopathic Scoliosis: A Pilot Randomized Controlled Trial

| **Section/Topic** | **Item No.** | **Checklist item** | **Reported on page No.** |
| --- | --- | --- | --- |
| **Title and abstract** | **1a** | Identification as a randomised trial in the title | 1 |
|  | **1b** | Structured summary of trial design, methods, results, and conclusions (for specific guidance see CONSORT for abstracts) | 2 |
| **Introduction: Background and objectives** | **2a** | Scientific background and explanation of rationale | 3 |
|  | **2b** | Specific objectives or hypotheses | 3 |
| **Methods: Trial design** | **3a** | Description of trial design (such as parallel, factorial) including allocation ratio | 4 |
|  | **3b** | Important changes to methods after trial commencement (such as eligibility criteria), with reasons | N/A - no important methodological changes after trial commencement |
| **Methods: Participants** | **4a** | Eligibility criteria for participants | 5 |
|  | **4b** | Settings and locations where the data were collected | 4 |
| **Methods: Interventions** | **5** | The interventions for each group with sufficient details to allow replication, including how and when they were actually administered | 6-7; Appendices 1-2 |
| **Methods: Outcomes** | **6a** | Completely defined pre-specified primary and secondary outcome measures, including how and when they were assessed | 7-8 |
|  | **6b** | Any changes to trial outcomes after the trial commenced, with reasons | N/A - no changes to pre-specified outcomes |
| **Methods: Sample size** | **7a** | How sample size was determined | 5 |
|  | **7b** | When applicable, explanation of any interim analyses and stopping guidelines | N/A - no interim analyses or stopping guidelines |
| **Methods: Randomisation - Sequence generation** | **8a** | Method used to generate the random allocation sequence | 5 |
|  | **8b** | Type of randomisation; details of any restriction (such as blocking and block size) | 5 |
| **Methods: Randomisation - Allocation concealment mechanism** | **9** | Mechanism used to implement the random allocation sequence (such as sequentially numbered containers), describing any steps taken to conceal the sequence until interventions were assigned | 5 |
| **Methods: Randomisation - Implementation** | **10** | Who generated the random allocation sequence, who enrolled participants, and who assigned participants to interventions | 5 |
| **Methods: Blinding** | **11a** | If done, who was blinded after assignment to interventions (for example, participants, care providers, those assessing outcomes) and how | 5 |
|  | **11b** | If relevant, description of the similarity of interventions | 6 |
| **Methods: Statistical methods** | **12a** | Statistical methods used to compare groups for primary and secondary outcomes | 8 |
|  | **12b** | Methods for additional analyses, such as subgroup analyses and adjusted analyses | N/A - no subgroup, adjusted, or other additional analyses |
| **Results: Participant flow (a diagram is strongly recommended)** | **13a** | For each group, the numbers of participants who were randomly assigned, received intended treatment, and were analysed for the primary outcome | 9; Figure 1 |
|  | **13b** | For each group, losses and exclusions after randomisation, together with reasons | 9; Figure 1 |
| **Results: Recruitment** | **14a** | Dates defining the periods of recruitment and follow-up | 4 |
|  | **14b** | Why the trial ended or was stopped | N/A - the trial was completed as planned |
| **Results: Baseline data** | **15** | A table showing baseline demographic and clinical characteristics for each group | 8; Table 1 |
| **Results: Numbers analysed** | **16** | For each group, number of participants (denominator) included in each analysis and whether the analysis was by original assigned groups | 9-10; Tables 2-6 |
| **Results: Outcomes and estimation** | **17a** | For each primary and secondary outcome, results for each group, and the estimated effect size and its precision (such as 95% confidence interval) | 9-10; Tables 2-6 |
|  | **17b** | For binary outcomes, presentation of both absolute and relative effect sizes is recommended | N/A - all outcomes were continuous |
| **Results: Ancillary analyses** | **18** | Results of any other analyses performed, including subgroup analyses and adjusted analyses, distinguishing pre-specified from exploratory | N/A - no ancillary analyses were performed |
| **Results: Harms** | **19** | All important harms or unintended effects in each group (for specific guidance see CONSORT for harms) | 7 and 9 |
| **Discussion: Limitations** | **20** | Trial limitations, addressing sources of potential bias, imprecision, and, if relevant, multiplicity of analyses | 14 |
| **Discussion: Generalisability** | **21** | Generalisability (external validity, applicability) of the trial findings | 14 |
| **Discussion: Interpretation** | **22** | Interpretation consistent with results, balancing benefits and harms, and considering other relevant evidence | 11-14 |
| **Other information: Registration** | **23** | Registration number and name of trial registry | 2 |
| **Other information: Protocol** | **24** | Where the full trial protocol can be accessed, if available | 16 |
| **Other information: Funding** | **25** | Sources of funding and other support (such as supply of drugs), role of funders | 16 |

Note: Page references correspond to the accompanying revised 19-page manuscript (Manuscript_English_FINAL_CONSORT.docx). Figure, table, and appendix references are included where applicable. N/A indicates that the item was not applicable to this trial.
